# Outcome and Prognostic Factors of Colorectal Endoscopic Submucosal Dissection in Patients Aged Over 75 Years

**DOI:** 10.1002/jgh3.70299

**Published:** 2025-11-10

**Authors:** Sota Nakagami, Takaaki Yoshikawa, Satoshi Okawa, Takeshi Mori, Naoya Osuki, Eito Kawaguchi, Mayu Nagae, Kumi Itami, Shinichiro Odo, Momoko Iketani, Sonoka Katsuyama, Kazuki Osawa, Ryo Itou, Kosuke Iwano, Shigeharu Nakano, Shunjiro Azuma, Toshihiro Morita, Kenshiro Hirohashi, Atsushi Yamauchi, Tadayuki Kou, Shujiro Yazumi

**Affiliations:** ^1^ Department of Gastroenterology and Hepatology, Kitano Hospital Tazuke Kofukai Medical Research Institute Osaka Japan

**Keywords:** colorectal tumors, elderly patients, endoscopic submucosal dissection, nutritional status, prognosis

## Abstract

**Objectives:**

We aimed to evaluate the safety and efficacy, and prognostic factors of colorectal endoscopic submucosal dissection (ESD) in patients aged ≥ 75 years.

**Methods:**

We retrospectively collected cases of colorectal ESD performed between January 2008 and December 2023. Patients were divided into two groups: elderly (≥ 75 years) and nonelderly (< 75 years). We compared clinicopathological characteristics, clinical outcomes, overall survival (OS), and disease‐specific survival (DSS) between the groups. We also analyzed factors associated with OS in the elderly group.

**Results:**

A total of 523 patients with 548 lesions were enrolled. Among them, 168 patients with 175 lesions were classified as elderly, and 355 patients with 373 lesions were in the nonelderly group. No significant differences were found in en bloc resection, curative resection, or complication rates between the two groups (*p* = 1.000, 0.703, and 0.583, respectively). The OS of the elderly group was significantly worse (*p* < 0.001), while DSS did not differ significantly (*p* = 0.155). A high Charlson comorbidity index and low prognostic nutritional index were associated with poorer OS in the elderly group.

**Conclusions:**

Colorectal ESD is a safe and effective treatment for patients aged ≥ 75 years. Indication for colorectal ESD should be carefully determined based on comorbidities and nutritional status to improve outcomes in this population.

## 1 Introduction

Colorectal cancer is the third most common cancer and the second leading cause of cancer‐related deaths worldwide [1]. Resection of precursor lesions and early‐stage cancers can reduce colorectal cancer‐related mortality and morbidity [2]. Endoscopic submucosal dissection (ESD) enables en bloc resection regardless of tumor size and facilitates accurate pathological evaluation. ESD is less invasive than surgical treatment and is considered appropriate for even older patients in terms of quality of life [3]. Despite improvements in technique, colorectal ESD still requires considerable experience owing to the risk of severe complications, such as perforation [4]. In older patients, comorbidities may affect outcomes, making prognostic evaluation important when considering ESD. Several studies have reported the safety and efficacy of colorectal ESD in older patients [3, 5, 6, 7, 8, 9]. However, factors associated with long‐term prognosis after ESD in older patients remain unclear. It also remains unknown whether additional surgery after noncurative resection prolongs their lives. Additionally, it is desirable to develop a nomogram predicting the survival of colorectal ESD in the elderly, based on the prognostic factors.

In this study, we evaluated the safety and efficacy of colorectal ESD in older patients. We also aimed to detect prognostic factors and develop a nomogram on the risk of survival after colorectal ESD in the elderly.

## 2 Methods

### 2.1 Patients

This retrospective cohort study was conducted at a single institution, the Medical Research Institute KITANO HOSPITAL (Osaka, Japan), and included patients who underwent colorectal ESD between January 2008 and December 2023. Patients were divided into two groups: elderly (aged ≥ 75 years) and nonelderly (aged < 75 years) [10]. Inclusion criteria were ESD for early colorectal cancer, adenoma, serrated lesions, and sporadic tumors with ulcerative colitis, and recurrent tumors after previous endoscopic resection. Exclusion criteria were ESD for neuroendocrine tumors, ulcerative colitis‐associated neoplasia, benign subepithelial lesions, and hybrid ESD. The American Society of Anesthesiologists physical status (ASA‐PS), body mass index (BMI), the original Charlson comorbidity index (CCI), and prognostic nutritional index (PNI) were used to assess patients' physical and nutritional conditions [11]. The original CCI was used to assess the severity of comorbidities [12]. PNI, an indicator of nutritional status, was calculated from blood test results as follows: PNI = 10 × serum albumin (g/dL) + 0.005 × total lymphocyte count (/μL) [13]. Each ESD session was counted separately for the same patient if the metachronous lesion was resected. However, if multiple lesions were resected in a single session, only one entry was included per patient.

### 2.2 ESD Procedures

All patients orally ingested 2 L of hypertonic polyethylene glycol solution (Niflec, Ajinomoto Co. Inc., Tokyo, Japan or EA Pharma Co. Ltd. Tokyo, Japan) for bowel preparation prior to colonoscopy. All patients were sedated with midazolam, propofol, or dexmedetomidine combined with pentazocine. Antispasmodics, butylscopolamine or glucagon, were used as needed. All cases of ESD procedures were performed under sedation and not under general anesthesia.

ESD was performed using the DualKnife (KD‐650L, 2012–2015), DualKnife J (KD‐655L, 2015–2023), or hook knife (KD‐620QR), all from Olympus (Tokyo, Japan). A solution consisting of 0.4% sodium hyaluronate (MucoUp; Boston Scientific, Marlborough, MA, USA) or glycerol (Hisiceol; Nipro, Osaka, Japan) was injected into the submucosa using a 25‐gauge injection needle (01961; Top Corp., Tokyo, Japan) or DualKnife J. Hemostatic forceps (Coagrasper, FD410LR, Olympus) (RAICHO2, RC2200‐2SC, KANEKA MEDICAL PRODUCTS, Osaka, Japan) were used for prophylactic coagulation of blood vessels and hemostasis of intraoperative bleeding. High‐frequency generators included the ICC200 (2006–2015), VIO200D (2015–2018), and VIO3 (2018–2023), all from Erbe Elektromedizin GmbH (Tübingen, Germany). All procedures were performed by or under the supervision of experienced endoscopists who had each performed over 50 colorectal ESDs. Endoscopic closure of post‐ESD defects depended on the decision of the endoscopist.

### 2.3 Histopathological Assessment

All resected specimens were stretched, pinned, fixed in 10% buffered formalin, and cut into 2‐mm slices. Specimens were examined histopathologically by pathologists at our hospital. Lesions were classified as adenoma, serrated lesion, Tis carcinoma (intramucosal adenocarcinoma), T1a carcinoma (adenocarcinoma with shallow submucosal invasion < 1000 μm), or T1b carcinoma (adenocarcinoma with deep submucosal invasion ≥ 1000 μm). The depth of the submucosal invasion was determined according to the General Rules for Clinical and Pathological Studies on Cancer of the Colon, Rectum, and Anus outlined by the Japanese Society for the Colon and Rectum (JSCCR) [14]. Criteria for curative resection were as follows: the lateral and vertical margins of the specimen were free of cancer, no poorly differentiated components, no lymphovascular invasion, a submucosal invasion depth < 1000 μm, and grade 1 budding [15]. Additional colectomy with lymph node dissection was performed based on the current guidelines.

### 2.4 Short‐Term Outcomes

Short‐term outcomes included en bloc resection, curative resection, operative time, days of hospitalization, and complications. We defined en bloc resection as the resection of the entire lesion in one piece, as confirmed endoscopically [16]. Procedure time was measured as the period from the first mucosal incision to complete lesion removal from the large intestine. Postoperative bleeding was defined as the presence of hematochezia and a decrease in hemoglobin of ≥ 2.0 g/dL [17]. Perforation during the procedure was defined as intraoperative perforation, whereas its occurrence after ESD completion was defined as delayed perforation [18]. Post‐ESD coagulation syndrome (PECS) was defined as localized abdominal tenderness with either fever (≥ 37.6°C) or inflammatory response (leukocytosis ≥ 10 000/μL or raised C‐reactive protein ≥ 0.5 mg/dL), without definite evidence of perforation, occurring ≥ 6 h after ESD [19].

### 2.5 Follow‐Up and Long‐Term Outcomes

According to the JSCCR guidelines, patients who underwent curative resection received a follow‐up colonoscopy 12 months post‐ESD, with subsequent follow‐up management dependent on the attending physicians [15]. In cases of noncurative resection, with or without additional surgery, abdominal ultrasonography and computed tomography were periodically performed in addition to colonoscopy. Local recurrence was defined as recurrence at the original resection site. Distant recurrence was defined as the occurrence of colorectal metastasis associated with the initial tumor. Survival time was calculated from the date of colorectal ESD to either the date of death or the most recent confirmation of survival. These surveys were conducted between July 2024 and September 2024. For analysis of long‐term outcomes, cases of discontinuation of ESD and followed up for < 6 months were excluded.

### 2.6 Statistical Analysis

Data are presented as medians with ranges. The Fisher's exact test was used to compare categorical data, and the Mann–Whitney *U* test was used to compare continuous data. Overall survival (OS) and disease‐specific survival (DSS) were determined using the Kaplan–Meier method and assessed using the log‐rank test. The Cox proportional hazards analysis was performed to identify factors associated with OS. We used the CCI and PNI as explanatory variables in the multivariate analysis and selected other explanatory variables according to previous reports [20, 21, 22]. All statistical analyses were performed using EZR (version 1.61; Saitama Medical Center, Jichi Medical University, Saitama, Japan) [23]. A *p* < 0.05 was considered statistically significant. A nomogram was created from the Cox proportional hazards analysis, using the rms packages in R software (version 4.5.1; r‐project.org; R Foundation for Statistical Computing, Vienna, Austria).

### 2.7 Study Approval

Informed consent was obtained through opt‐out forms on our website (https://kitano.bvits.com/rinri/publish_document.aspx?ID=1095). This study was approved by the ethics committee of the Medical Research Institute, KITANO HOSPITAL (2409005) and conducted in accordance with the Declaration of Helsinki.

## 3 Results

### 3.1 Patient Characteristics

**FIGURE 1:**
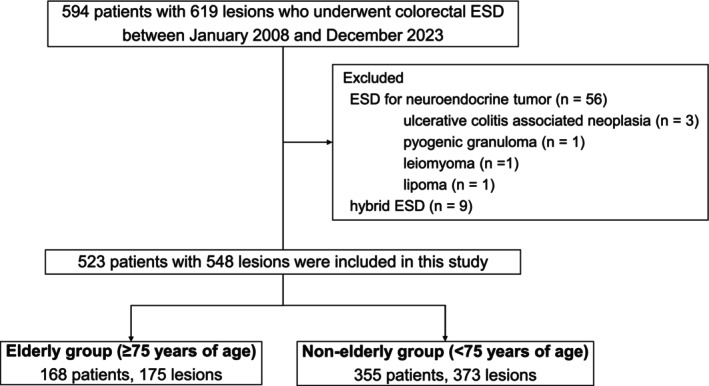
The flowchart of enrollment and selection of patients and lesions in this study. ESD, endoscopic submucosal dissection.

A flowchart of the enrollment and selection of patients for this study is shown in Figure 1. Between January 2008 and December 2023, 594 patients with 619 lesions underwent ESD. After excluding 71 patients with 71 lesions (ESD for neuroendocrine tumors, *n* = 56; ulcerative colitis‐associated neoplasia, *n* = 3; pyogenic granuloma, *n* = 1; leiomyoma, *n* = 1; lipoma, *n* = 1; hybrid ESD, *n* = 9), 523 patients with 548 lesions were included. Of these, 168 patients with 175 lesions comprised the elderly group, and 355 patients with 373 lesions formed the nonelderly group. Patient characteristics are summarized in Table 1. The median ages (range) of the groups were 79 (75–92) and 65 (37–74) years, respectively. The elderly group had a worse median ASA‐PS score (2 [1–3]; *p* < 0.001) and median PNI (48.9 [30.2–63.8]; *p* < 0.001). The proportion of patients using antithrombotic drugs was significantly higher in the elderly group (51%; *p* = 0.002). There were no significant differences in BMI and the proportion of patients with a CCI ≥ 3 (*p* = 0.250 and 0.106, respectively).

**TABLE 1:** Clinical characteristics of patients.

	Elderly group	Nonelderly group	*p*
Patients, *n*	168	355	
Lesions, *n*	175	373	
Age, years, median (range)	79 (75–92)	65 (37–74)	
Sex, *n* (%), male/female	94 (56.0)/74 (44.0)	209 (58.9)/146 (41.1)	0.570
ASA‐PS, median (range)	2 (1–3)	2 (1–3)	< 0.001
ASA‐PS, *n* (%), 1–2/3–4	118 (70.2)/50 (29.8)	291 (82.0)/64 (18.0)	0.003
BMI, kg/m^2^, median (range)	22.5 (14.4–30.3)	22.9 (13.8–36.7)	0.250
Comorbidities, *n* (%)			
Hypertension	94 (56.0)	148 (41.7)	0.003
Dyslipidemia	52 (31.0)	100 (28.2)	0.537
Diabetes	24 (14.3)	57 (16.1)	0.698
Renal disease	26 (15.5)	24 (6.8)	0.002
Liver disease	5 (3.0)	9 (2.5)	0.776
Use of antithrombotic drugs, *n* (%)	51 (30.4)	65 (18.3)	0.002
CCI, median (range)	1 (0–8)	0 (0–8)	0.011
CCI ≥ 3, *n* (%)	25 (14.9)	35 (9.9)	0.106
Total lymphocyte count, /μL, median (range)	1510 (370–3790)	1640 (430–4330)	0.002
Serum albumin levels, g/dL, median (range)	4.1 (2.6–4.7)	4.3 (2.2–5.2)	< 0.001
PNI, median (range)	48.9 (30.2–63.8)	50.9 (28.4–69.7)	< 0.001
PNI < 46, *n* (%)	51 (30.4)	44 (12.4)	< 0.001

Abbreviations: ASA‐PS, American Society of Anesthesiologists physical status; BMI, body mass index; CCI, Charlson comorbidity index; PNI, prognostic nutritional index.

### 3.2 Pathological Characteristics

The pathological features of the lesions are summarized in Table 2. No significant differences were observed between the groups in tumor location, macroscopic type, and size (*p* = 0.426, 0.258, and 0.840, respectively). A total of 85 lesions (48.6%) in the elderly group and 151 (40.5%) in the nonelderly group were diagnosed as adenocarcinomas, with no significant difference (*p* = 0.079). Procedure discontinuation occurred in 8 lesions (4.6%) in the elderly group and 17 lesions (4.6%) in the nonelderly group. The cause of discontinuation was muscle‐retracting sign positive in 24 lesions and perforation in 1 lesion. Among the lesions of discontinuation, surgery was performed in 21 lesions, chemotherapy in 1 lesion, and 3 lesions were followed conservatively (Table S1).

**TABLE 2:** Pathological characteristics of the lesions.

	Elderly group	Nonelderly group	*p*
Lesions, *n*	175	373	
Location, *n* (%), colon/rectum	126 (72.0)/49 (28.0)	255 (68.4)/118 (31.6)	0.426
Macroscopic type, *n* (%), superficial/protruded	133 (76.0)/42 (24.0)	265 (71.0)/108 (29.0)	0.258
Tumor size, mm, median (range)	25.5 (7–114)	25.5 (3–105)	0.840
Histology, *n* (%)			0.373
Adenoma	78 (44.6)	187 (50.1)	
Serrated lesion	4 (2.3)	18 (4.8)	
Tis carcinoma	56 (32.0)	90 (24.1)	
T1a carcinoma	12 (6.9)	26 (7.0)	
T1b carcinoma	17 (9.7)	35 (9.4)	
Procedure discontinuation	8 (4.6)	17 (4.6)	

Abbreviations: Tis carcinoma, intramucosal adenocarcinoma; T1a carcinoma, adenocarcinoma with shallow submucosal invasion < 1000 μm; T1b carcinoma, adenocarcinoma with deep submucosal invasion ≥ 1000 μm.

### 3.3 Short‐Term Outcomes

Short‐term outcomes are summarized in Table 3. The mean procedure time was 88 (12–415) min in the elderly group and 90 (10–450) min in the nonelderly group (*p* = 0.848). The en bloc resection rates were 95.4% in both groups (*p* = 1.000), and curative resection rates were 84.0% and 85.3%, respectively (*p* = 0.703). The rates of intraoperative perforation (4.6%) and postoperative bleeding (4.0%) in the elderly group were not significantly different from those in the nonelderly group. However, one case of ESD‐related death occurred in the elderly group due to a large intramural colonic hematoma. An 82‐year‐old man who had been prescribed edoxaban underwent ESD for a rectosigmoid 25 mm Is lesion. Significant fibrosis in the submucosal layer caused difficulty in dissection and resulted in a microperforation. Clip closure was performed after en bloc resection; the histology was intramucosal tubular adenocarcinoma (tub1). The patient developed an intramural hematoma and generalized peritonitis by postoperative Day 4, resulting in death [24]. There were no significant differences in overall complication rates between the two groups (13.7% vs. 12.1%, *p* = 0.583).

**TABLE 3:** Short‐term outcomes of colorectal ESD.

	Elderly group	Nonelderly group	*p*
Lesions, *n*	175	373	
Procedure time, min, median (range)	88 (12–415)	90 (10–450)	0.848
En bloc resection, *n* (%)	167 (95.4)	356 (95.4)	1.000
Curative resection, *n* (%)	147 (84.0)	318 (85.3)	0.703
Hospital stay, day, median (range)	7 (3–52)	7 (2–23)	0.278
Total complication, *n* (%)	24 (13.7)	45 (12.1)	0.583
Delayed bleeding, *n* (%)	7 (4.0)	15 (4.0)	1.000
Intraoperative perforation, *n* (%)	8 (4.6)	16 (4.3)	1.000
Delayed perforation, *n* (%)	2 (1.1)	1 (0.3)	0.240
PECS, *n* (%)	6 (3.4)	13 (3.5)	1.000
ESD‐related death, *n* (%)	1 (0.6)	0 (0)	0.319

Abbreviations: ESD, endoscopic submucosal dissection; PECS, post‐ESD coagulation syndrome.

### 3.4 Long‐Term Outcomes

**FIGURE 2:**
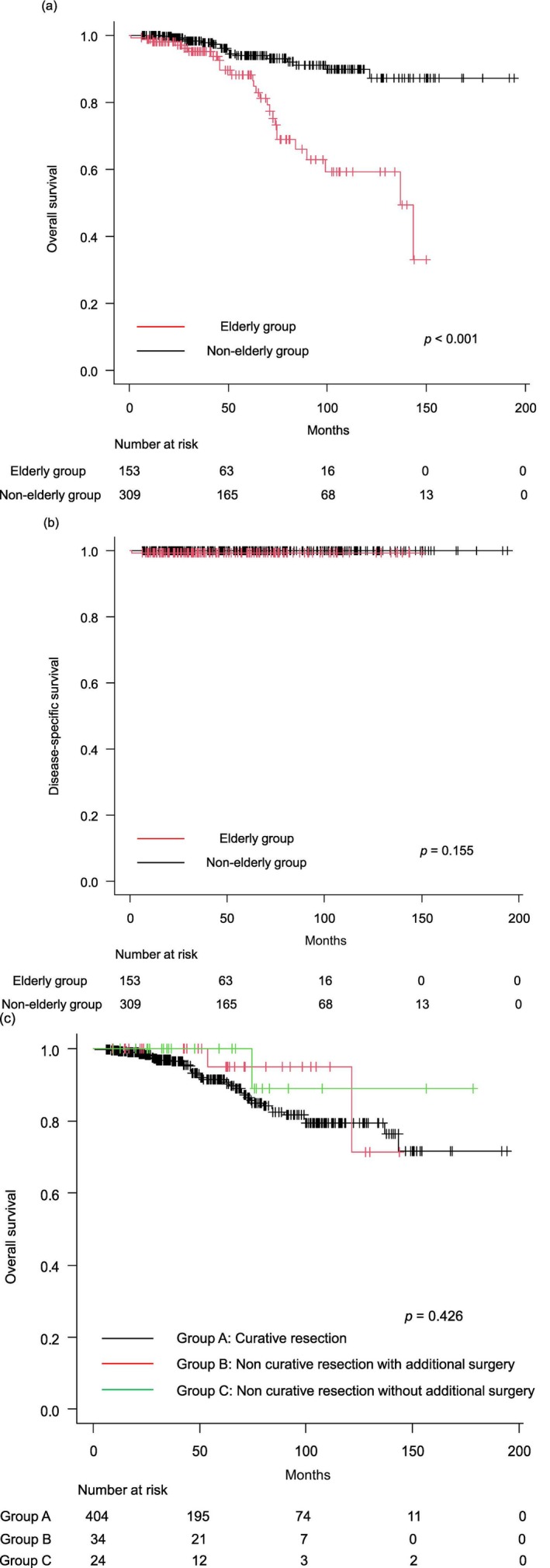
(a) Kaplan–Meier curves of overall survival for the elderly and nonelderly group. (b) Kaplan–Meier curves of disease‐specific survival for the elderly and nonelderly group. (c) Kaplan–Meier curves of overall survival between curability for the elderly and nonelderly group.

Long‐term outcomes are summarized in Table 4. Cases of ESD discontinuation (23 patients) and followed up for < 6 months (38 patients) were excluded; 153 patients in the elderly group and 309 patients in the nonelderly group were analyzed. The median follow‐up periods in the elderly and nonelderly groups were 38.7 (0.8–152.1) and 56.9 (6.6–197.1) months, respectively (*p* = 0.004). The proportion of patients who underwent additional surgery after noncurative resection was significantly lower in the elderly group (35.0%, 7/20) compared to the nonelderly group (71.1%, 27/38) (*p* = 0.012). However, local and distant recurrences were observed only in the nonelderly group. Both groups underwent salvage resection (re‐ESD and surgery, respectively). No recurrence was observed in the elderly group. During the follow‐up period, 26 patients (15.0%) in the elderly group and 17 patients (5.5%) in the nonelderly group died. The causes of death are summarized in Table 5. In the elderly group, only one patient died of ESD‐related death, as mentioned in the short‐term outcomes. Other causes of death included other malignant tumors (*n* = 11), pneumonia (*n* = 3), renal failure (*n* = 1), pulmonary embolism (*n* = 1), and unknown causes (*n* = 9). The 3‐year OS of the elderly group (95.1%) was significantly worse than that of the nonelderly group (98.3%; *p* < 0.001) (Figure 2a). However, the 3‐year DSS rate did not differ significantly between the two groups (99.3% vs. 100%, *p* = 0.155) (Figure 2b). Also, OS between curability did not differ significantly (*p* = 0.426) (Figure 2c).

**TABLE 4:** Long‐term outcomes of colorectal ESD.

	Elderly group	Nonelderly group	*p*
Patients, *n*	153	309	
Follow‐up period, months, median (range)	38.7 (0.8–152.1)	56.9 (6.6–197.1)	0.004
Curability			
Curative resection, *n* (%)	133 (86.9)	271 (87.7)	0.028
Noncurative resection with additional surgery, *n* (%)	7 (4.6)	27 (8.7)	
Noncurative resection without additional surgery, *n* (%)	13 (8.5)	11 (3.6)	
Local recurrence, *n* (%)	0	1 (0.3)	1.000
Distant recurrence, *n* (%)	0	1 (0.3)	1.000
Total deaths, *n* (%)	26 (17.0)	17 (5.5)	< 0.001
Deaths caused by colorectal tumor, *n* (%)	1 (0.6)	0	0.320
3‐Year overall survival, % (95% CI)	95.1 (89.3–97.8)	98.3 (95.6–99.4)	< 0.001
3‐Year disease‐specific survival, % (95% CI)	99.3 (95.5–99.9)	100	0.155

Abbreviations: CI, confidence interval; ESD, endoscopic submucosal dissection.

**TABLE 5:** Cause of death during follow‐up.

	Elderly group (*n* = 26/153)	Nonelderly group (*n* = 17/309)
ESD‐related death	1	
Other malignant tumors		
Lung cancer	11	14
Pancreatic cancer	3	4
Gastric cancer	2	2
Hepatocellular carcinoma	1	3
Breast cancer	1	1
Prostate cancer	1	2
Leukemia/malignant lymphoma	1	1
Oral cancer	1	
Esophageal cancer	1	1
Pneumonia	3	
Renal failure	1	
Pulmonary embolism	1	
Unknown causes	9	3

Abbreviation: ESD, endoscopic submucosal dissection.

### 3.5 Prognostic Factors and Predictive Nomogram for OS in the Elderly Group

To determine the factors associated with OS in the elderly group, we used the Cox proportional hazards model (Table 6). In univariate analysis, a high ASA‐PS (≥ 3), high CCI (≥ 3), and low PNI were significantly associated with poor OS (*p* = 0.016, < 0.001, and < 0.001, respectively). Carcinoma tended to be associated with poor OS (*p* = 0.061), while noncurative resection was not significantly associated with poor OS (*p* = 0.139). Multivariate analysis indicated that high CCI (hazard ratio [HR]: 3.81; 95% confidence interval [CI]: 1.52–9.54; *p* = 0.004) and low PNI (HR: 0.87; 95% CI: 0.78–0.96; *p* = 0.006) remained associated with poor OS. Among the nonelderly group, high CCI was associated with poor OS in univariate analysis (HR: 22.4; 95% CI: 7.74–64.8; *p* < 0.001) (Table S2). In contrast, low PNI was not associated with poor OS (HR: 0.93; 95% CI: 0.83–1.04; *p* = 0.213).

**TABLE 6:** Prognostic factors for overall survival in the elderly group.

Variables	Univariate analysis		Multivariate analysis	
	HR (95% CI)	*p*	HR (95% CI)	*p*
Age ≥ 80 years^a^	1.02 (0.45–2.29)	0.966		
Sex male	2.20 (0.82–5.89)	0.116		
ASA‐PS ≥ 3	2.68 (1.20–5.98)	0.016		
BMI	0.91 (0.79–1.04)	0.159		
CCI ≥ 3	5.00 (2.06–12.2)	< 0.001	3.81 (1.52–9.54)	0.004
PNI	0.85 (0.77–0.93)	< 0.001	0.87 (0.78–0.96)	0.006
PNI < 46	0.44 (0.19–1.01)	0.054		
Carcinoma^b^	2.22 (0.96–5.12)	0.061		
Noncurative resection	0.22 (0.03–1.63)	0.139		
Noncurative resection without additional surgery	0.46 (0.06–3.43)	0.450		

Abbreviations: ASA‐PS, American Society of Anesthesiologists physical status; BMI, body mass index; CCI, Charlson comorbidity index; CI, confidence interval; HR, hazard ratio; PNI, prognostic nutritional index.

^a^ Reference group is age 75–79 years.

^b^ Pathological findings of intramucosal carcinoma or submucosal invasive carcinoma.

**FIGURE 3:**
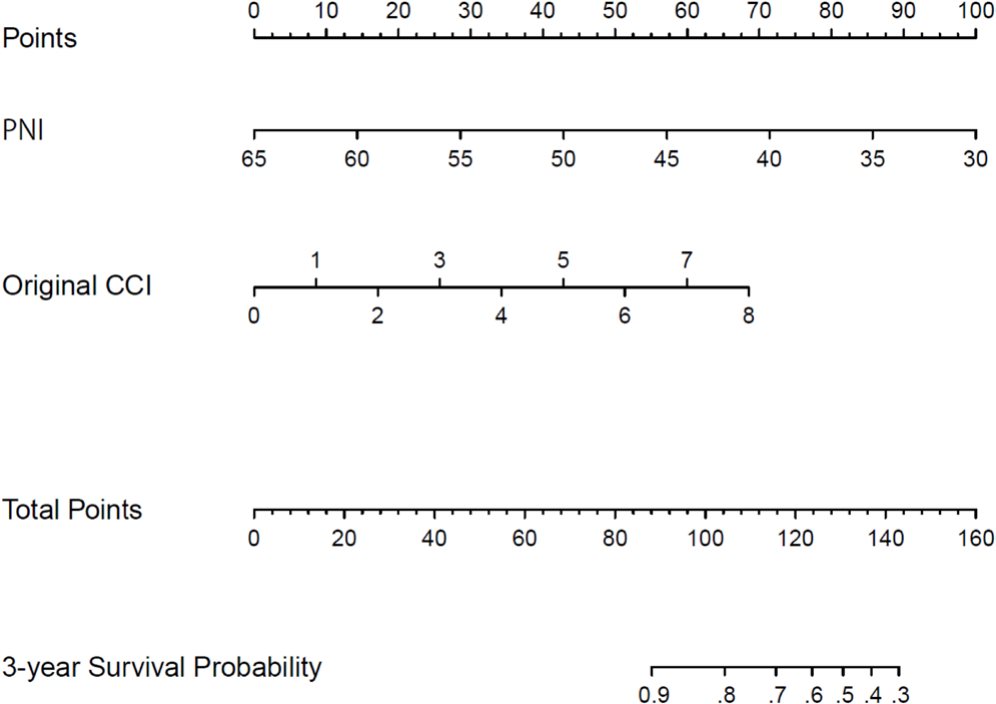
Prognostic nomogram for predicting overall survival of the elderly group. The nomogram can assign the probability of survival by adding up the scores identified on the points scale for each variable.

Based on the findings, the model that incorporated these predictors was developed and presented as a predictive nomogram for 3‐year OS in the elderly group (Figure 3).

## 4 Discussion

This study evaluated the safety, efficacy, and long‐term prognosis of colorectal ESD in patients aged ≥ 75 years, as well as prognostic factors for OS in this population.

The en bloc and curative resection rates in the elderly group were 95.4% and 84.9% in this study. These findings are consistent with those of previous reports, which reported en bloc resection rates of 88%–97% and curative resection rates of 89%–90.4% [18, 25, 26], supporting the validity of our ESD procedures. Notably, these rates did not differ significantly between the elderly and nonelderly groups, suggesting that the curability of colorectal ESD might be independent of age.

The total complication rate in this study was 12.6% (69/548), including an interoperative perforation rate of 4.4% (24/548), which is comparable to previously reported rates [18, 25, 26]. The result also proved that our ESD procedures were safe. No significant differences in intraoperative perforation or postoperative bleeding were observed between the two groups, although the rate of antithrombotic drug use was significantly higher in the elderly group. Therefore, our study suggests that ESD can be safely performed in patients aged ≥ 75 years. However, one case of a huge colonic intramural hematoma caused a fatal complication [24]. Owing to the high rate of antithrombotic drug use in the elderly, potentially fatal complications due to bleeding may occur.

The rate of additional surgery after noncurative resection was lower in the elderly group (35.0%, 7/20) compared to the nonelderly group (71.1%, 27/38), aligning with previous studies [6, 9]. In the present study, two patients experienced recurrence (one local and one distant) exclusively in the nonelderly group, and both patients underwent re‐ESD or surgery without subsequent recurrence. In the elderly group, noncurative resection was not a significant prognostic factor for OS. Therefore, additional surgery may not affect the prognosis of patients aged ≥ 75 years.

In contrast, we found that the CCI and PNI were associated with the prognosis of colorectal ESD in the elderly group. In recent studies, CCI and PNI have been suggested as prognostic factors for esophageal and gastric ESD [27, 28, 29, 30]. Previous studies have also pointed to the possibility of the CCI and nutritional status (geriatric nutritional risk index) as prognostic factors for colorectal ESD [20, 21, 22]. These findings suggest the need for cautious case selection for colorectal ESD in patients aged ≥ 75 years with multimorbidity and poor nutritional status. Our study also found that low CCI was associated with poor OS in the nonelderly group. However, there was hardly any study about the prognosis of colorectal ESD for nonelderly [31], it is still unknown whether our prognostic factors are peculiar problems to the elderly or not. In addition, we developed a prognostic nomogram for predicting OS of the elderly. This nomogram may serve as an exploratory tool to support patient counseling and optimization of comorbidities and nutritional status.

This study has some limitations. First, this was a single‐center retrospective analysis. Second, follow‐up intervals varied and depended on the attending physicians. Third, approximately half of the lesions were benign (adenomas or serrated lesions). Fourth, we did not include discontinued cases in our long‐term analyses. Fifth, the developed nomogram was created using only two variables, CCI and PNI. However, the prognosis for elderly patients is influenced by numerous complex factors such as functional status, frailty, and social factors. It is considered necessary to create nomograms incorporating additional factors and to verify external validity.

## 5 Conclusions

Colorectal ESD appears to be a safe and effective treatment in patients aged ≥ 75 years. However, the elderly group had a shorter OS. High CCI and low PNI were identified as prognostic factors for poor OS in this population. CCI and PNI should be considered when determining the appropriateness of colorectal ESD in patients aged over 75 years.

## Conflicts of Interest

The authors declare no conflicts of interest.

## Supporting information


**Table S1:** Characteristics and management for procedure discontinuation lesions.

**Table S2:** Prognostic factors for overall survival in the non‐elderly group.

## Data Availability

The data that support the findings of this study are available on request from the corresponding author. The data are not publicly available due to privacy or ethical restrictions.
