# Supplementary material for: Outcome and Prognostic Factors of Colorectal Endoscopic Submucosal Dissection in Patients Aged Over 75 Years
Source: JGH Open. 2025 Nov 10;9(11):e70299. doi: 10.1002/jgh3.70299 (PMC12602999; doi:10.1002/jgh3.70299)
Supplement: Supplementary file 1 — Table S1: Characteristics and management for procedure discontinuation lesions. [file JGH3-9-e70299-s001.docx]

**Supplementary Table 1.** Characteristics and management for procedure discontinuation lesions

|  | **Elderly group**  **(n = 8)** | **Non-elderly group**  **(n = 17)** |
| --- | --- | --- |
| Location, n (%), Colon/Rectum | 5/3 | 11/6 |
| Macroscopic type, n (%), Superficial/Protruded | 2/6 | 4/13 |
| Tumor size, mm, median (range) | 30 (15-70) | 30 (12-40) |
| Cause of procedure discontinuation  Muscle-retracting sign positive  Perforation | 8  0 | 16  1 |
| Additional treatment  Surgery  　Chemotherapy  　Follow-up | 5  1  2 | 16  1 |
| Histology, n (%)  Adenoma  Tis carcinoma  　T1a carcinoma  　T1b carcinoma  T2 carcinoma  T3 carcinoma  No neoplastic lesion  Unknown | 1  2  1  1  3 | 1  3  2  5  3  1  1  1 |

Tis carcinoma, intramucosal adenocarcinoma; T1a carcinoma, adenocarcinoma

with shallow submucosal invasion <1000 µm; T1b carcinoma, adenocarcinoma with deep submucosal invasion ≥1000 µm; T2 carcinoma, adenocarcinoma

with muscularis propria invasion; T3 carcinoma, adenocarcinoma with pericolorectal tissues invasion through the muscularis propria
