# Supplementary material for: Outcome and Prognostic Factors of Colorectal Endoscopic Submucosal Dissection in Patients Aged Over 75 Years
Source: JGH Open. 2025 Nov 10;9(11):e70299. doi: 10.1002/jgh3.70299 (PMC12602999; doi:10.1002/jgh3.70299)
Supplement: Supplementary file 2 — Table S2: Prognostic factors for overall survival in the non‐elderly group. [file JGH3-9-e70299-s002.docx]

**Supplementary Table 2.** Prognostic factors for overall survival in the non-elderly group

| **Variables** | **Univariate analysis** | |
| --- | --- | --- |
|  | **HR (95% CI)** | ***p* value** |
| Sex Male | 3.62 (1.04–12.6) | 0.043 |
| ASA-PS ≥3 | 7.61 (2.81–20.6) | <0.001 |
| BMI | 0.88 (0.77–1.01) | 0.068 |
| CCI ≥3 | 22.4 (7.74–64.8) | <0.001 |
| PNI | 0.93 (0.83–1.04) | 0.213 |
| PNI <46 | 0.54 (0.15–1.90) | 0.337 |
| Carcinoma † | 0.87 (0.34–2.27) | 0.781 |
| Non-curative resection | 0.82 (0.19–3.58) | 0.790 |
| Non-curative resection  without additional surgery | 0.00 (0.00-Inf) | 0.998 |

† Pathological findings of intramucosal carcinoma or submucosal invasive carcinoma

ASA-PS, American Society of Anesthesiologists physical status; BMI, body mass index; CCI, Charlson comorbidity index; PNI, prognostic nutritional index; HR, hazard ratio; CI, confidence interval
